# Randomized phase II study of SOX+B-mab versus SOX+C-mab in patients with previously untreated recurrent advanced colorectal cancer with wild-type KRAS (MCSGO-1107 study)

**DOI:** 10.1186/s12885-021-08690-y

**Published:** 2021-08-23

**Authors:** Yujiro Nishizawa, Naotsugu Haraguchi, Hirotoshi Kim, Yoshihito Ide, Ken Nakata, Shu Okamura, Toshihiro Kudo, Taroh Satoh, Mamoru Uemura, Chu Matsuda, Tsunekazu Mizushima, Kohei Murata, Yuichiro Doki, Hidetoshi Eguchi

**Affiliations:** 1 Department of Gastroenterological Surgery, Osaka General Medical Center, 3-1-56 Mandaihigashi Sumiyoshi-ku, Osaka, 558-8558 Japan; 2 Department of Gastroenterological Surgery, Graduate School of Medicine, Osaka University, 2-2-E2 Yamadaoka, Suita, Osaka, 565-0871 Japan; 3 Department of Gastroenterological Surgery, Osaka International Cancer Institute, 3-1-69 Otemae, Chuo-ku, Osaka, 541-8567 Japan; 4 Department of Surgery, Rinku General Medical Center, 2-23 Rinku Orai-kita, Izumisano, Osaka, 598-8577 Japan; 5 Department of Surgery, Japan Community Healthcare Organization Osaka Hospital, 4-2-78 Fukushima, Fukushima-ku, Osaka, 553-0003 Japan; 6 Department of Surgery, Sakai City Medical Center, 1-1-1 Ebaraji-cho, Nishi-ku, Sakai, Osaka, 593-8304 Japan; 7 Department of Surgery, Suita Municipal Hospital, 5-7 Kishibeshinmachi, Suita, Osaka, 564-8567 Japan; 8 Department of Surgery, Kansai Rosai Hospital, 3-1-69 Inabasou, Amagaski-shi, Hyogo 660-8511 Japan

**Keywords:** Cetuximab, Chemotherapy, Colorectal cancer, Early tumor shrinkage, KRAS, Oxaliplatin

## Abstract

**Background:**

Although chemotherapy for metastatic colorectal cancer (mCRC) has improved, the standard chemotherapy regimens for patients with RAS wild-type mCRC remain debated. This study aimed to compare S-1 and oxaliplatin (SOX) + bevacizumab (B-mab) with SOX + cetuximab (C-mab) in patients with previously untreated recurrent advanced CRC with wild-type KRAS.

**Methods:**

This randomized phase II, open-label, multicenter study compared the efficacy and safety of SOX+B-mab with SOX+C-mab in patients with previously untreated advanced CRC with wild-type KRAS. Between February 2012 and October 2016, 45 patients were enrolled.

**Results:**

Overall response rates were 59.1 and 43.5% (*p* = 0.29) and disease control rates were 90.9 and 91.3% (*p* = 0.96) in the SOX+B-mab and SOX+C-mab groups, respectively. Median overall survival (OS) was 25.3 and 15.5 months (HR = 0.607, *p* = 0.167) and median progression-free survival (PFS) were 11.7 and 5.5 months (HR = 0.558, *p* = 0.077) in the SOX+B-mab and SOX+C-mab groups, respectively. The OS and PFS of patients with early tumor shrinkage (ETS) were not significantly different in the SOX+B-mab group. However, they were significantly better when ETS was ≥20 in the SOX+C-mab group (*p* = 0.032 and *p* = 0.003, respectively).

**Conclusions:**

The efficacy and safety of SOX+B-mab and SOX+C-mab for wild-type KRAS recurrent advanced CRC as first-line chemotherapy were almost the same. Consideration of the treatment strategy based on ETS may improve patient prognosis, especially in patients receiving the SOX+C-mab regimen.

**Trial registration:**

UMIN Clinical Trials Registry (UMIN000006706).

Date of registration: NOV/11/2011.

URL of trial registry record:

https://upload.umin.ac.jp/cgi-open-bin/ctr_e/ctr_view.cgi?recptno=R000007920

**Supplementary Information:**

The online version contains supplementary material available at 10.1186/s12885-021-08690-y.

## Background

Colorectal cancer (CRC) is the third most common malignancy in men and the second most common in women, and it ranks third in terms of incidence but second in terms of mortality in both sexes worldwide. In 2018, over 1.8 million new CRC cases and about 900,000 deaths were estimated to occur, accounting for about 10% of all cancer cases and deaths [1]. Approximately 20–25% of patients with CRC show synchronous metastases, and an additional 20–25% of patients will develop metastases after curative resection [2, 3]. Although clinical outcomes in patients with metastatic colorectal cancer (mCRC) have improved over the last decade in particular and the median OS for patients with mCRC is over 30 months and more than double that of 20 years ago, the standard-of-care chemotherapy regimens for patients with RAS wild-type mCRC remain debated [4]. A phase III trial of irinotecan/5-FU/leucovorin (FOLFIRI) or oxaliplatin/5-FU/leucovorin (mFOLFOX6) with bevacizumab or cetuximab for patients with KRAS wild-type untreated mCRC (CALGB/SWOG80405), in which the primary endpoint was OS, showed no significant difference in the combination of chemotherapy with cetuximab or bevacizumab, but it showed a trend toward longer OS in cetuximab-treated patients versus bevacizumab-treated patients with FOLFOX [5]. A randomized, open-label, phase III trial of FOLFIRI plus cetuximab versus FOLFIRI plus bevacizumab as first-line treatment for patients with mCRC (FIRE-3) did not meet its primary endpoint: objective tumor response. Although there was no significant difference in progression-free survival (PFS), a difference in OS with a benefit of 3.7 months in cetuximab-treated patients was observed in the KRAS exon 2 wild-type population [6]. A post-hoc analysis of tumor dynamics in the final RAS wild-type subgroup showed a 7.5-month benefit in OS with cetuximab (hazard ratio [HR] 0.7) [7].

S-1 is an oral anticancer agent that combines tegafur with two modulators: gimeracil and oteracil potassium [8]. A phase III trial designed to validate the non-inferiority of S-1 and oxaliplatin (SOX) plus bevacizumab to mFOLFOX6 plus bevacizumab (SOFT study) in terms of PFS in patients with mCRC who had not previously received chemotherapy demonstrated the non-inferiority of SOX plus bevacizumab to mFOLFOX6 plus bevacizumab [9]. Updated OS analyses of the SOFT study demonstrated that SOX plus bevacizumab is non-inferior to mFOLFOX6 plus bevacizumab in terms of PFS(10.2 months vs 10.2 months), and the authors concluded that SOX plus bevacizumab is considered an effective regimen for first-line chemotherapy in patients with mCRC and can be used instead of mFOLFOX6 plus bevacizumab [10].

No study has compared the difference of SOX plus bevacizumab and SOX plus cetuximab as a first-line treatment for untreated mCRC in the RAS-wild population. The present randomized phase II study aimed to compare SOX plus bevacizumab with SOX plus cetuximab in patients with previously untreated recurrent advanced CRC with wild-type KRAS.

## Methods

### Ethics statements

This Multi-center Clinical Study Group of Osaka, Colorectal Cancer Treatment Group (MCSGO)-1107 (UMIN000006706) study was conducted in accordance with the ethical principles of the Declaration of Helsinki and in compliance with Japanese ethical guidelines for clinical studies. The study protocol was approved by the institutional review board of each participating institution, and written informed consent was obtained from all patients before enrolment.

### Study design and patients

This phase II, randomized, open-label, multicenter study evaluated the efficacy and safety of SOX with bevacizumab or cetuximab in patients with previously untreated, unresectable, locally advanced, or metastatic CRC with wild-type KRAS. The KRAS status of exon 2 (codons 12/13) was verified by local polymerase chain reaction. Eligible patients were aged ≥20 years with previously untreated, locally advanced, histologically proven, unresectable or metastatic CRC. If postoperative adjuvant chemotherapy was administered, registration was approved as long as at least 180 days had passed since the last dose. In addition, patients had an Eastern Cooperative Oncology Group performance status of 0 or 1, ≥1 measurable lesion per RECIST version 1.1 (v1.1) [11], life expectancy > 3 months, and adequate organ function. Patients were excluded if they had prior adjuvant chemotherapy including oxaliplatin, active malignancy requiring treatment, active autoimmune disease, active infection requiring systemic treatment, continuous systemic steroid treatment, interstitial lung disease, active hepatitis B virus infection, active non-infectious pneumonitis, or pregnancy.

Participants were randomly assigned in a 1:1 ratio to receive either bevacizumab or cetuximab by using a validated computer system (Meditrix Corporation, Tokyo, Japan). Randomization was performed centrally with the use of the minimization method and the following stratification factors: postoperative adjuvant chemotherapy, liver metastasis, and institution. Independent central investigators used a web-based system for enrolment, which then automatically assigned patients to each cohort.

### Procedures

All enrolled patients received intravenous oxaliplatin (130 mg/m^2^) with 7.5 mg/kg of bevacizumab (cohort A) on day 1 or intravenous oxaliplatin (130 mg/m^2^) with 400 mg/m^2^ (250 mg/m^2^ after two courses) of cetuximab (cohort B) and TS-1 orally at a dose of 80–120 mg/day (body surface area [BSA] < 1.25 m^2^, 80 mg; BSA 1.25–1.50 m^2^, 100 mg; BSA ≥1.50 m^2^, 120 mg) that was divided into two daily doses for 14 days followed by 7 days of rest until disease progression, unacceptable toxicity, or study withdrawal. Patients who discontinued treatment for reasons other than progression were followed until loss to follow-up or withdrawal of consent. Tumor response was assessed every 9 weeks per RECIST v1.1. Survival was assessed every 9 weeks during follow-up. Adverse events (AEs) were graded by investigators according to the National Cancer Institute CTCAE (version 4) [12] and were monitored throughout the study.

### Outcomes

The primary end point was the overall response rate (ORR; the proportion of patients with complete response [CR] or partial response [PR]) assessed per RECIST v1.1. Secondary end points were disease control rate (DCR; the proportion of patients with CR + PR + stable disease for ≥24 weeks before progressive disease [PD]), OS (time from first study treatment to death as a result of any cause), PFS (time from first study treatment to first confirmed PD or death, whichever occurred first), time-to-treatment failure (TTF), treatment completion rate, rate of R0 resection induction, timing of therapeutic effect, safety, and tolerability. Patients without confirmed death at the data cutoff were censored at the date of the last follow-up.

### Sample size calculation

The additional response rate of the combination of bevacizumab or cetuximab to SOX therapy was assumed to be about 30%. The threshold response rate in each cohort was set at 50%, and the expected response rate was set at 80%, with α = 0.05 (one-sided) and 1-β = 0.9. The required number of cases was calculated to be 21 cases. The target sample size was set at 25 in each cohort, with a total sample size of 50, considering some exclusions and dropouts.

### Statistical analyses

A chi-square test was provided for the response rate and DCR (per RECIST v1.1) in both cohorts. Kaplan-Meier estimates were provided for PFS and OS. HRs and their confidence interval (CIs) were calculated using a Cox proportional hazards model for multivariate analysis. Early tumor shrinkage (ETS) was defined as a ≥ 20% decrease in the sum of the longest diameters of RECIST target lesions at 3 months as compared with the baseline. The depth of response (DpR) was defined as the percentage of tumor shrinkage, based on the longest diameters as compared with the baseline. The efficacy and safety analysis populations included all patients in both cohorts. Safety was assessed using descriptive analyses. Statistical analyses were performed using JMP Pro 14.1.0 software (SAS Institute, Cary, NC, USA).

## Results

### Patients

**Fig. 1:**
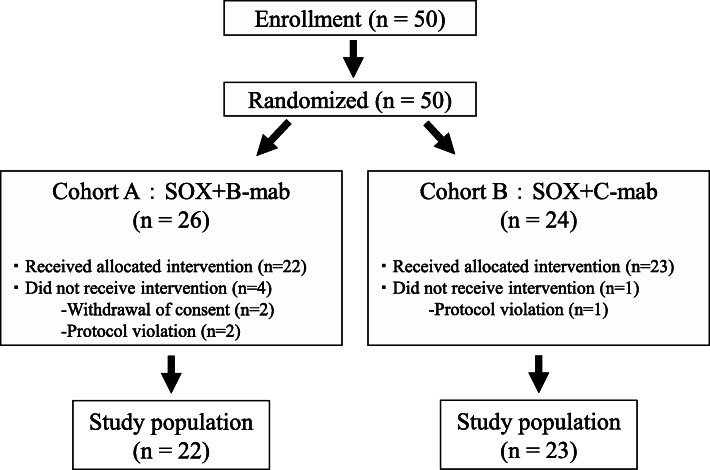
Flow chart. Forty-five patients were eligible for study inclusion

Between February 27, 2012, and October 31, 2016, a total of 50 patients with advanced mCRC from 11 institutions belonging to the MCSGO were enrolled in this study. Figure 1 shows a flow chart of study patients. Among the 50 patients, 3 violated the protocol and 2 withdrew consent; therefore, 45 patients were eligible for study inclusion. Table 1 summarizes the patient and tumor characteristics. These characteristics (were well balanced between the two groups. All patients were locally advance cancers (T3 or deeper). In both groups, the rates of primary tumor resected at study entry were comparable (*p* = 0.235) and approximately 80% of tumors were located in the left side of the colon.

**Table 1:** Patient and tumor characteristics treated with SOX+B-mab or SOX+C-mab

Characteristics	SOX+B-mab (*n* = 22)	SOX+C-mab (*n* = 23)	Total (*n* = 45)
Age, median (range), year	67 (49–79)	66 (40–79)	66 (40–79)
Sex			
Male	14 (66.7)	15 (65.2)	29 (64.4)
Female	8 (33.3)	8 (34.8)	16 (35.6)
ECOG performance status			
0	17 (71.4)	18 (78.3)	35 (77.8)
1	4 (23.8)	4 (17.4)	8 (17.8)
2	1 (4.8)	1 (4.3)	2 (4.4)
TNM at study entry			
T T3 / T4a / T4b	10 / 11 / 1	14 / 4 / 5	24 / 15 / 6
N N0 / N1/ N2a / N2b	5 / 7 / 7 / 3	5 / 6 / 9 / 3	10 / 13 / 16 / 6
M M1a / M1b / M1c	12 / 8 / 2	8 / 10 / 5	20 / 18 / 7
Primary tumor resected at study entry			
Yes	17 (77.3)	14 (60.9)	31 (68.9)
No	5 (22.7)	9 (39.1)	14 (31.1)
Metastatic sites at start of treatment			
Liver only	5 (22.7)	4 (17.4)	9 (20.0)
Liver	14 (63.6)	15 (65.2)	29 (64.4)
Lung	4 (18.2)	8 (34.8)	12 (26.7)
Lymph nodes	15 (68.2)	15 (65.2)	30 (66.7)
Peritoneum	3 (13.6)	5 (21.7)	8 (17.8)
Others	1 (4.5)	3 (13.0)	4 (8.9)
Colorectal cancer location at diagnosis			
Left	17 (77.3)	19 (82.6)	36 (81.4)
Right	5 (21.7)	4 (17.4)	9 (18.6)

*ECOG* Eastern Cooperative Oncology Group

### Efficacy

Median follow up was 19.9 months (range, 1.5–55.4 months) for patients in the SOX+B-mab group and 12.0 months (range, 0.8–59.4 months) for patients in the SOX+C-mab group. The median number of treatment courses was five in both groups (*p* = 0.837; Supplementary Table 1). As shown in Table 2, the ORR for the SOX+B-mab group was 59.1%, whereas that for the SOX+C-mab group was 43.5% (*p* = 0.29). In both groups, no patient had CR. The DCR for the SOX+B-mab group was 90.9%, whereas that of the SOX+C-mab group was 91.3% (*p* = 0.96). Conversion surgery (equal to R0 resection induction) was performed in 18.2% of patients in the SOX+B-mab group and 4.3% of patients in the SOX+C-mab group (*p* = 0.19). TTFs were 4.61 months (range, 0.95–14.3 months) in the SOX+B-mab group and 4.38 months (range, 0.53–12.5 months) in the SOX+C-mab group (*p* = 0.785; Supplementary Table 1).

**Table 2:** Best overall response to treatment in the SOX+B-mab and SOX+C-mab population

Outcomes (RECIST v1.1)	SOX+B-mab (*n =* 22)	SOX+C-mab (*n =* 23)	Total (*n =* 45)
Overall response rate (CR + PR)	13 (59.1%)	10 (43.5%)	23 (51.1%), *P =* 0.29
Disease control rate (CR + PR + SD)	20 (90.9%)	21 (91.3%)	41 (91.1%), *P =* 0.96
Complete response (CR)	0 (0.0%)	0 (0.0%)	0 (0.0%)
Partial response (PR)	13 (59.1%)	10 (43.5%)	23 (51.1%)
Stable disease (SD)	7 (31.8%)	11 (47.8%)	18 (40.0%)
Progressive disease (PD)	1 (4.5%)	1 (4.3%)	2 (4.4%)
Could not be evaluated (NE)	1 (4.5%)	1 (4.3%)	2 (4.4%)
Conversion surgery	4 (18.2%)	3 (13.0%)	7 (15.6%)
R0 resection	4 (18.2%)	1 (4.3%)	5 (11.1%), *P =* 0.19
R2 resection	0	2 (8.7%)	2 (4.4%)

**Fig. 2:**
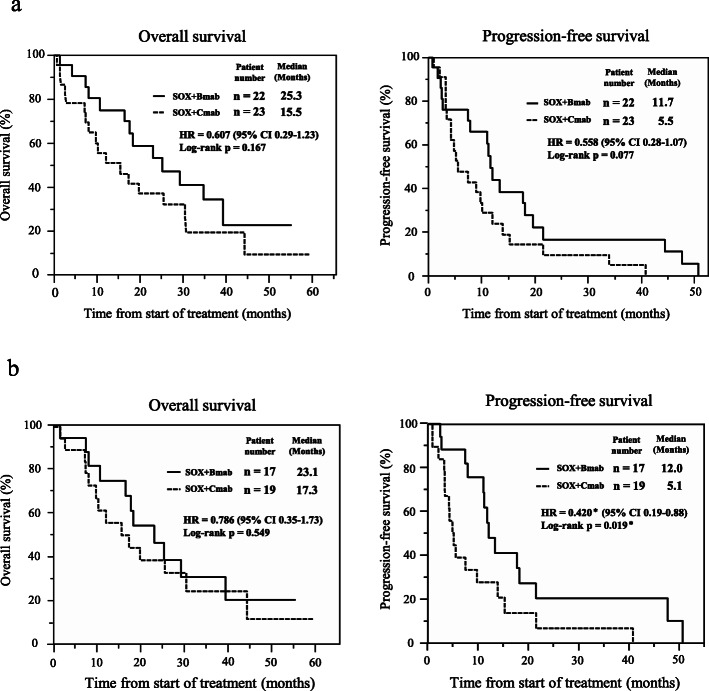
Kaplan-Meier curves for overall survival and progression-free survival for all patients (a) and patients with left-sided colorectal cancer (b). Solid black line: SOX+B-mab, dotted black line: SOX+C-mab. * indicates < 0.05

Figure 2 shows the Kaplan-Meier curves for OS and PFS for all patients or patients with only left-sided colorectal cancer. For all patients, median OS were 25.3 months (95% CI: 16.5–39.4 months) in the SOX+B-mab group and 15.5 months (95% CI: 7.30–30.4 months, *p* = 0.167) in the SOX+C-mab group. Median PFS were 11.7 months (95% CI: 7.37–18.2 months) in the SOX+B-mab group and 5.5 months (95% CI: 3.36–10.1 months, *p* = 0.077) in the SOX+C-mab group. According to the Kaplan-Meier curves limited to left-sided colon cancer, OS was not significantly different between the groups (*p* = 0.55), but PFS was significantly better in the SOX+B-mab group (12.0 months [95% CI: 7.9–21.4 months]) than in the SOX+C-mab group (5.1 months [95% CI: 3.3–9.7. months, *p* = 0.019]).

**Fig. 3:**
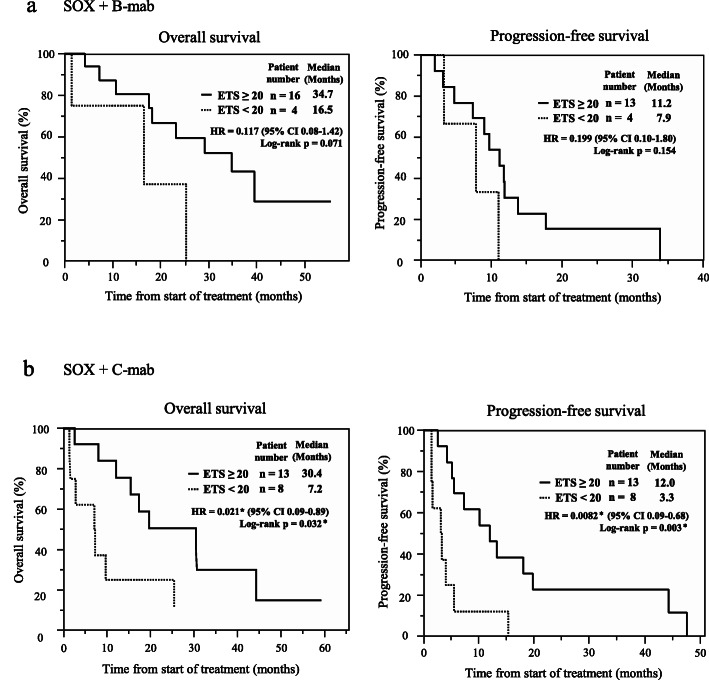
Kaplan-Meier curves for overall survival and progression-free survival for SOX+B-mab group (a) and SOX+C-mab group (b) classified by ETS. Solid black line: ETS ≥20, dotted black line: ETS < 20. * indicates < 0.05

Next, ETS was assessed in each group, and the cutoff point of ETS was set to 20%. ETS of the SOX+B-mab group was 37.5%, whereas that of the SOX+C-mab group was 30.1% (*p* = 0.42, Supplementary Table 2). Figure 3 shows Kaplan-Meier curves for OS and PFS of the SOX+B-mab and SOX+C-mab groups classified by the presence or absence of ETS. In the SOX+B-mab group, OS and PFS were not significantly different with and without ETS. However, in the SOX+C-mab group, patients with ETS had significantly better OS (30.4 months [95% CI: 8.0–44.3 months, *p* = 0.032]) and PFS (12.0 months [95% CI: 5.1–19.7 months, *p* = 0.003]) than those without ETS.

Additionally, the DpR of the SOX+B-mab group was 40%, whereas that of the SOX+C-mab group was 30.1% (*p* = 0.41, Supplementary Table 2). Supplementary Fig. 1 shows the waterfall plot of the best change in size of target lesions. Days and courses of timing of therapeutic effect were not significantly different between the two groups (Supplementary Table 3). Univariate and multivariate analyses for OS and PFS showed no significant independent prognosis factors (Supplementary Table 4 and 5).

### Safety

The treatment-related AEs are summarized in Table 3. All grades of AEs occurred in 20/22 patients (90.9%) in the SOX+B-mab group and 23/23 patients (100%) in the SOX+C-mab group. Grade ≥ 3 AEs occurred in 10/22 patients (45.6%) in the SOX+B-mab group and 11/23 of patients (47.8%) in the SOX+C-mab group. The most common AEs were peripheral sensory neuropathy in both groups, and allergic reaction and paronychia were distinctive AEs in the SOX+C-mab group. Skin and subcutaneous tissue disorders were also AEs characterized in the SOX+C-mab group. One patient in the SOX+B-mab group had grade 4 malaise, but no patients died of treatment-related AEs. AEs that caused discontinuation of treatment occurred in seven patients (31.8%) in the SOX+B-mab group and nine patients (39.1%) in the SOX+C-mab group (*p* = 0.61).

**Table 3:** Adverse events (treatment-related)

Adverse events	SOX+B-mab (*n =* 22)		SOX+C-mab (*n =* 23)	
	All grades (%)	≧Grade 3 (%)	All grades (%)	≧Grade 3 (%)
**Hematologic adverse events**				
Leucopenia	3 (13.6)	0 (0.0)	1 (4.3)	0 (0.0)
Anemia	7 (31.8)	1 (4.8)	6 (26.1)	1 (4.3)
Thrombocytopenia	6 (27.2)	1 (4.8)	8 (34.8)	3 (13.0)
Hyperbilirubinemia	8 (36.4)	2 (9.1)	4 (17.4)	0 (0.0)
Hypoalbuminemia	6 (27.2)	1 (4.8)	7 (30.4)	0 (0.0)
Aspartate aminotransferase increased	6 (27.2)	0 (0.0)	8 (34.8)	1 (4.3)
Alanine aminotransferase increased	6 (27.2)	0 (0.0)	9 (39.1)	1 (4.3)
Hypomagnesemia	1 (4.8)	0 (0.0)	1 (4.3)	0 (0.0)
Hyperkalemia	1 (4.8)	0 (0.0)	3 (13.0)	0 (0.0)
**Nonhematologic adverse events**				
Mucositis oral	4 (18.2)	0 (0.0)	6 (26.1)	0 (0.0)
Nausea	3 (13.6)	0 (0.0)	5 (21.7)	0 (0.0)
Vomiting	1 (4.8)	0 (0.0)	5 (21.7)	1 (4.3)
Diarrhea	5 (22.7)	1 (4.8)	11 (43.5)	2 (8.7)
Anorexia	9 (40.9)	2 (9.1)	9 (39.1)	2 (8.7)
Fatigue	3 (13.6)	2 (9.1)	5 (21.7)	0 (0.0)
Malaise	7 (31.8)	1 (4.8)	9 (39.1)	0 (0.0)
Allergic reaction	0 (0.0)	0 (0.0)	2 (8.7)	0 (0.0)
Peripheral sensory neuropathy	18 (81.8)	2 (9.1)	16 (69.6)	0 (0.0)
Peripheral motor neuropathy	3 (13.6)	0 (0.0)	2 (8.7)	0 (0.0)
Palmar-plantar erythrodysesthesia syndrome	2 (9.1)	0 (0.0)	4 (17.4)	0 (0.0)
Proteinuria	3 (13.6)	0 (0.0)	3 (13.0)	0 (0.0)
Hypertension	3 (13.6)	0 (0.0)	2 (8.7)	1 (4.3)
Skin and subcutaneous tissue disorders	1 (4.8)	0 (0.0)	12 (52.2)	0 (0.0)
Paronychia	0 (0.0)	0 (0.0)	10 (43.5)	0 (0.0)
**Total**	20 (90.9)	10 (45.6)	23 (100)	11 (47.8)

## Discussion

This is the first randomized phase II, open-label, multicenter study to compare the efficacy and safety of SOX+bevacizumab with SOX+cetuximab in patients with previously untreated recurrent advanced colorectal cancer with wild-type KRAS. The ORR, the primary endpoint, was not significantly different between the two study groups (*p* = 0.29). However, the treatment effect tended to be better in the SOX+B-mab group than in the SOX+C-mab group. Although there was no significant difference between the two groups in PFS and OS, these outcomes tended to be better in the SOX+B-mab group than in the SOX+C-mab group; there were differences of about 10 months for OS and about 6 months for PFS in both groups. The SOFT study reported a median PFS (assessed RECIST) of 10.2 months and an ORR of 61.5% in the SOX+B-mab group [10], which were similar to the results in the SOX+B-mab group of our study (PFS, 11.7 months; ORR, 59.1%). Multivariate analyses for OS and PFS with variables including age, sex, tumor sidedness, treatment regimen, location of metastasis showed no significant independent prognosis factors. Additionally, in our study, there was no difference in the number of treatment courses, TTF, and rate of discontinuation due to side effects between the groups.

Because the SOX+B-mab and SOX+C-mab groups comprised about 80% of left-sided colon cancer in our study, we analyzed OS and PFS in only patients with left-sided colorectal cancer. Although OS was not different between the groups, PFS was significantly better in the SOX+B-mab group than in the SOX+C-mab group. In general, the significance of anti-epidermal growth factor receptor (EGFR) antibodies has been proven in wild-type RAS left-sided colorectal cancer [13]. The ESMO guidelines recommend the use of anti-EGFR antibodies as a treatment for wild-type RAS left-sided colon cancer [14]. In the FIRE-3 study, although there was no significant difference in PFS, there was a difference in OS with a benefit of 3.7 months in the C-mab-treated patients in the wild-type KRAS exon 2 population compared to B-mab-treated patients [7]. There are possibilities behind the differences in PFS in the SOX+C-mab and SOX+B-mab groups in patients with left-sided colorectal cancer. The first possibility is secondary or subsequent treatment after failure of this study regimen. In this study, we did not limit secondary or subsequent treatment. In fact, three patients (13.6%) in the SOX+B-mab group used a regimen that included cetuximab and nine patients (39.1%) of the SOX+C-mab group used a regimen that included bevacizumab in secondary or subsequent treatment. The second possibility is the number of treatment courses. In the SOFT study, the median number of treatment courses was eight. However, that of our study was five in both groups. This relatively shorter treatment course may affect the difference in PFS. The third possibility is dose intensity. Although AEs seemed to not be different in hematological events, non-hematological events of peripheral neuropathy and hypertension were high in the SOX+B-mab group. Allergic reactions and paronychia were distinctive, and skin and subcutaneous tissue disorders characterized AEs in the SOX+C-mab group. Notably, all grades of AEs in nausea, vomiting, and diarrhea were observed almost over double in the SOX+C-mab group. The combination of oral fluoropyrimidine with anti-EGFR agents is known to increase the risk of diarrhea [15]. The concomitant study of the MRC COIN trial showed that OxFU+cetuximab and OxCap+cetuximab were equivalent in terms of OS, ORR, and RRS(rate of radical surgeries). Nonetheless, PFS was longer with OxFU+cetuximab than with OxCap+cetuximab, and the authors described a possibility that the higher toxicity associated with ≥grade 3 nausea, diarrhea, and palmar-plantar erythema in OxCap+cetuximab led to greater dose reductions and a lower total dose of oxaliplatin [16]. These AEs possibly decrease the dose intensity in the combination of SOX with cetuximab, and it is necessary to assess dose intensity in a large-scale study.

This study have some limitations. A limitation of this study is that KRAS status was assessed only on exon 2 (codons 12/13). Evidence from the PRIME study and CRYSTAL study has shown that tumors with additional RAS mutations (exons 3 and 4 of KRAS and exons 2, 3, and 4 of NRAS) other than those in KRAS exon 2 display a lack of response to EGFR-targeting monoclonal antibodies [17, 18]. Furthermore, *BRAF* mutations are almost exclusively non-overlapping with *RAS* mutations and are reported to be negative predictive biomarkers for EGFR antibody therapy in patients with mCRC [19–21]. Final analysis of the randomized PEAK trial supports the importance of expanded RAS mutational analysis and showed longer median PFS and median OS for panitumumab versus bevacizumab in wild-type RAS and BRAF CRC [22]. In response to the results of these clinical trials, the ESMO consensus guideline recommends expanding *RAS* mutational analysis to at least KRAS exons 2, 3, and 4 (codons 12, 13, 59, 61, 117, and 146) and NRAS exons 2, 3, and 4 (codons 12, 13, 59, 61, and 117) alongside the assessment of tumor *BRAF* mutational status. The presence of these minor *RAS* and *BRAF* mutations may have affected the results of this study. Indeed, other *RAS* mutations were detected in 14.7 and 31% of evaluable tumors previously assessed to be wild-type KRAS exon 2 in the CRYSTAL study and in the OPUS study, respectively [17, 22]. The other limitation is sample size. We calculated sample size based on previous reports that the additional response rate of bevacizumab or cetuximab for SOX therapy was approximately 30%. In fact, the additional response rate was lower than expected. Accumulation of further cases remains likely to have significant results.

Recently, ETS and DpR have been focused on as prognostic factors for RFS and OS after first-line treatment of mCRC [6]. In our study, OS and PFS did not significantly differ between ETS < 20 and ETS ≥20 in the SOX+B-mab group. However, OS and PFS were significantly better in the ETS ≥20 group than in the ETS < 20 group among patients in the SOX+C-mab group. Anti-EGFR antibody drugs are reported to have a shorter TTR, better DpR, and more ETS than B-mab [23]. Patients with ETS in both groups had an OS > 30 months and PFS > 11 months, but the benefits of ETS to OS and PFS were significantly higher in the SOX+C-mab group than in the SOX+B-mab group. The assessment of ETS can be a powerful marker for prognosis even in patients receiving SOX with C-mab. When C-mab is used in combination with SOX, evaluation of ETS is indispensable, and if ETS is < 20 after 3 months, consideration of the treatment strategy including drug change may be useful for improving patient prognosis.

## Conclusions

The safety and efficacy of SOX+B-mab and SOX+C-mab for wild-type KRAS, recurrent advanced CRC as a first-line chemotherapy were almost the same, but they tended to be better in the SOX+B-mab group than in the SOX+C-mab group. ETS was more correlated with PFS in the SOX+C-mab group than in the SOX+B-mab group, and consideration of treatment strategy based on ETS may improve patient prognosis, especially in patients receiving the SOX+C-mab regimen.

## Supplementary Information

**Additional file 1: Supplementary Fig. 1.** Best percentage change in size of target lesions in the SOX+B-mab (a) and SOX+C-mab (b) population (Waterfall plot).

**Additional file 2: Supplemental Table.1.** Time to treatment Failure and Number of Treatment Courses.

**Additional file 3: Supplemental Table.2.** ETS and DpR.

**Additional file 4: Supplementary Table.3.** Timing of therapeutic effect.

**Additional file 5: Supplementary Table.4.** Univariate and multivariate analysis for overall survival.

**Additional file 6: Supplementary Table 5.**. Univariate and multivariate analysis for Progression-free survival.

## Data Availability

The datasets used and/or analyzed during the current study are available from the corresponding author on reasonable request.

## Abbreviations

mCRC: Metastatic colorectal cancer
SOX: S-1 and oxaliplatin
B-mab: Bevacizumab
C-mab: Cetuximab
ETS: Early tumour shrinkage
OS: Overall survival
FOLFIRI: Irinotecan/5-FU/leucovorin
FOLFOX: Oxaliplatin/5-FU/leucovorin
PFS: Progression-free survival
AEs: Adverse events
ORR: Overall response rat**e**
DCR: Disease control rate
TTF: Time to treatment failure
CI: Confidence interval
DpR: Depth of response
