# Supplementary material for: Randomized phase II study of SOX+B-mab versus SOX+C-mab in patients with previously untreated recurrent advanced colorectal cancer with wild-type KRAS (MCSGO-1107 study)
Source: BMC Cancer. 2021 Aug 23;21:947. doi: 10.1186/s12885-021-08690-y (PMC8381542; doi:10.1186/s12885-021-08690-y)
Supplement: Supplementary file 1 — Additional file 1: Supplementary Fig. 1. Best percentage change in size of target lesions in the SOX+B-mab (a) and SOX+C-mab (b) population (Waterfall plot). [file 12885_2021_8690_MOESM1_ESM.pptx]

## Slide 1
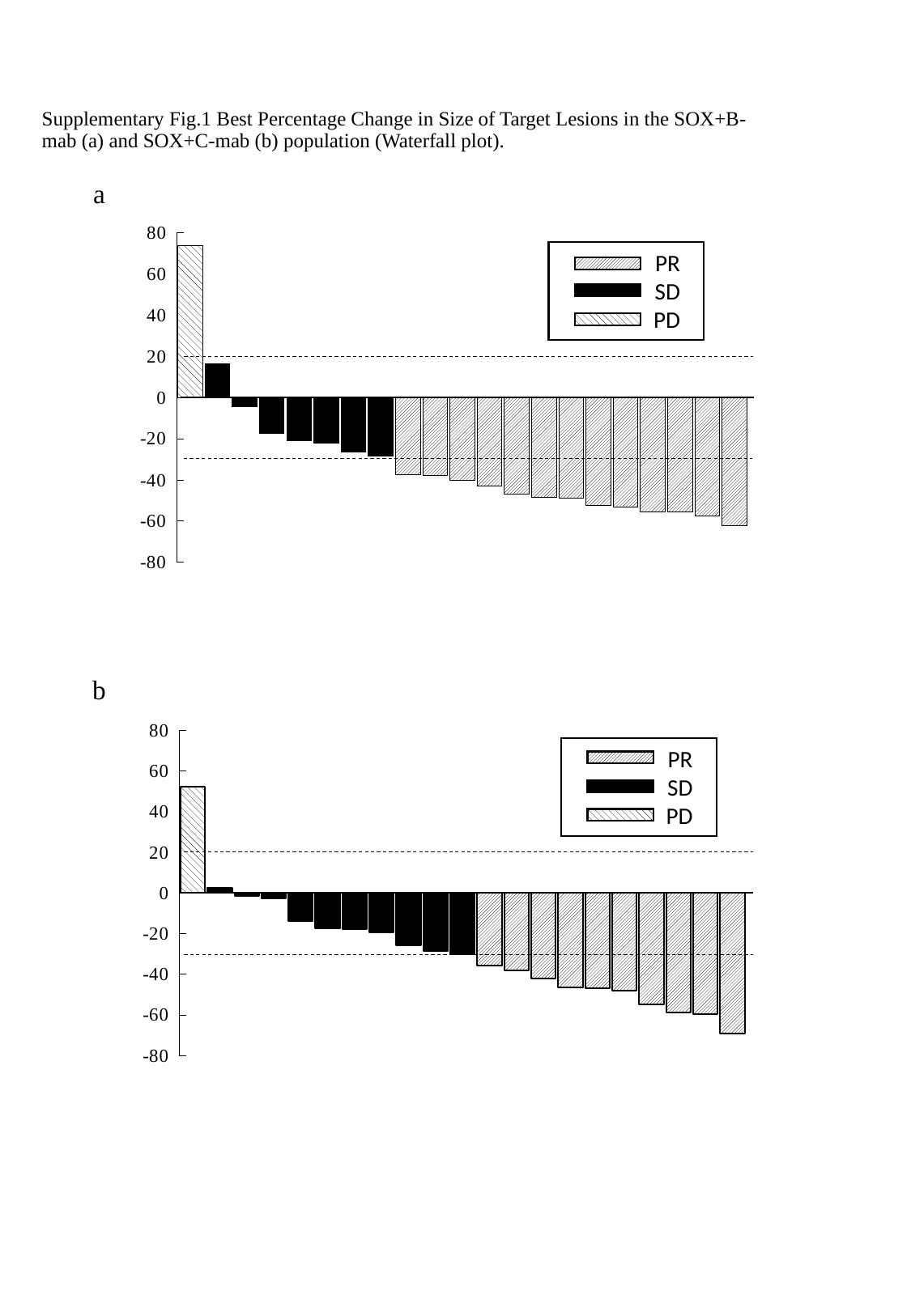

# Supplementary Fig.1 Best Percentage Change in Size of Target Lesions in the SOX+B-mab (a) and SOX+C-mab (b) population (Waterfall plot).
a
### Chart
| Category | |
|---|---|PR
SD
PD
b
### Chart
| Category | |
|---|---|PR
SD
PD
