# Supplementary material for: Randomized phase II study of SOX+B-mab versus SOX+C-mab in patients with previously untreated recurrent advanced colorectal cancer with wild-type KRAS (MCSGO-1107 study)
Source: BMC Cancer. 2021 Aug 23;21:947. doi: 10.1186/s12885-021-08690-y (PMC8381542; doi:10.1186/s12885-021-08690-y)
Supplement: Supplementary file 2 — Additional file 2: Supplemental Table.1. Time to treatment Failure and Number of Treatment Courses. [file 12885_2021_8690_MOESM2_ESM.docx]

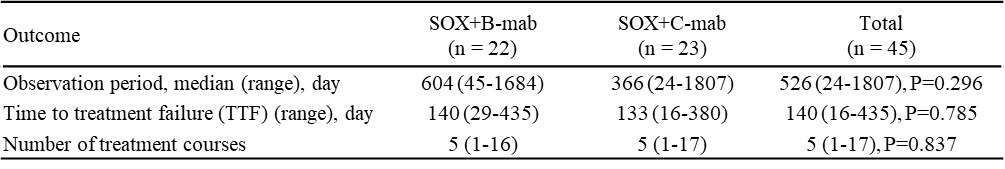


P=0.837

Number of treatment courses

0

5

10

15

SOX+B-mab

SOX+C-mab

Number of treatment courses

P=0.785

Time of treatment failure

0

50

100

150

200

250

300

350

400

450

SOX+B-mab

SOX+C-mab

Time of treatment failure

Supplemental Table.1 Time to treatment Failure and Number of Treatment Courses
