# Supplementary material for: Randomized phase II study of SOX+B-mab versus SOX+C-mab in patients with previously untreated recurrent advanced colorectal cancer with wild-type KRAS (MCSGO-1107 study)
Source: BMC Cancer. 2021 Aug 23;21:947. doi: 10.1186/s12885-021-08690-y (PMC8381542; doi:10.1186/s12885-021-08690-y)
Supplement: Supplementary file 3 — Additional file 3: Supplemental Table.2. ETS and DpR. [file 12885_2021_8690_MOESM3_ESM.docx]

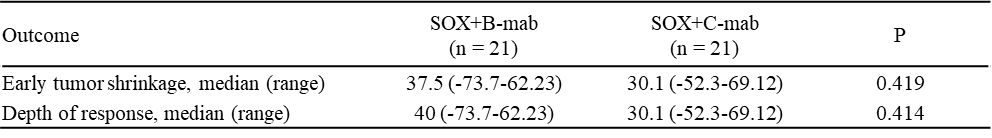


-75

-50

-25

0

25

50

75

Depth of response (%)

P=0.414

SOX+B-mab

SOX+C-mab

DpR

-75

-50

-25

0

25

50

75

P=0.419

SOX+B-mab

SOX+C-mab

ETS

Percentage of ETS (%)

Supplemental Table.2 ETS and DpR
