# Supplementary material for: Randomized phase II study of SOX+B-mab versus SOX+C-mab in patients with previously untreated recurrent advanced colorectal cancer with wild-type KRAS (MCSGO-1107 study)
Source: BMC Cancer. 2021 Aug 23;21:947. doi: 10.1186/s12885-021-08690-y (PMC8381542; doi:10.1186/s12885-021-08690-y)
Supplement: Supplementary file 4 — Additional file 4: Supplementary Table.3. Timing of therapeutic effect. [file 12885_2021_8690_MOESM4_ESM.docx]

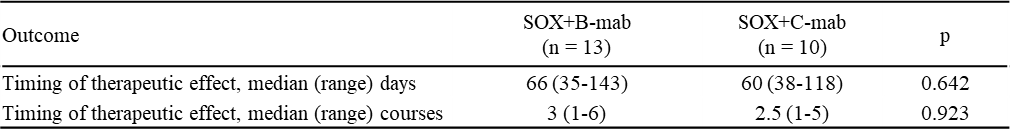


Timing of therapeutic effct (days)

20

40

60

80

100

120

140

160

Timing of therapeutic effect (days)

P = 0.642

SOX+B-mab

SOX+C-mab

P = 0.923

SOX+B-mab

SOX+C-mab

Timing of therapeutic effect (courses)

0

1

2

3

4

5

6

7

Timing of therapeutic effct (courses)

Supplementary Table.3 Timing of therapeutic effect
