# Supplementary material for: Randomized phase II study of SOX+B-mab versus SOX+C-mab in patients with previously untreated recurrent advanced colorectal cancer with wild-type KRAS (MCSGO-1107 study)
Source: BMC Cancer. 2021 Aug 23;21:947. doi: 10.1186/s12885-021-08690-y (PMC8381542; doi:10.1186/s12885-021-08690-y)
Supplement: Supplementary file 5 — Additional file 5: Supplementary Table.4. Univariate and multivariate analysis for overall survival. [file 12885_2021_8690_MOESM5_ESM.docx]

Supplementary Table.4

Univariate and multivariate analysis for overall survival

(Cox regression model)

| Clinicopathological factors | Univariate | | | Multivariate | | |
| --- | --- | --- | --- | --- | --- | --- |
|  | HR | 95 % CI | *p* value | HR | 95 % CI | *p* value |
| Age  (75≧ / 75<) | 1.488 | 0.4437 – 4.987 | 0.52 | 1.492 | 0.4379 – 5.083 | 0.5224 |
| Sex  （Male / Female) | 1.113 | 0.5234 – 2.368 | 0.7803 | 1.186 | 0.5515 – 2.553 | 0.6618 |
| Location of metastasis  (Liver only / The others) | 1.049 | 0.4247 – 2.59 | 0.9179 | 1.031 | 0.3758 – 2.831 | 0.9522 |
| Colorectal cancer location  (Right / Left) | 1.135 | 0.4554 – 2.83 | 0.7854 | 1.333 | 0.4721 – 3.763 | 0.5873 |
| Treatment regimen  （SOX+C-mab / SOX+B-mab） | 1.648 | 0.8052 – 3.372 | 0.1716 | 1.745 | 0.8318 – 3.659 | 0.1408 |

HR: Hazard ratio, CI: Confidence interval
