# Supplementary material for: Randomized phase II study of SOX+B-mab versus SOX+C-mab in patients with previously untreated recurrent advanced colorectal cancer with wild-type KRAS (MCSGO-1107 study)
Source: BMC Cancer. 2021 Aug 23;21:947. doi: 10.1186/s12885-021-08690-y (PMC8381542; doi:10.1186/s12885-021-08690-y)
Supplement: Supplementary file 6 — Additional file 6: Supplementary Table 5.. Univariate and multivariate analysis for Progression-free survival. [file 12885_2021_8690_MOESM6_ESM.docx]

Supplementary Table 5.

Univariate and multivariate analysis for Progression-free survival

(Cox regression model)

| Clinicopathological factors | Univariate | | | Multivariate | | |
| --- | --- | --- | --- | --- | --- | --- |
|  | HR | 95 % CI | *p* value | HR | 95 % CI | *p* value |
| Age  (75≧ / 75<) | 1.067 | 0.4138 – 2.749 | 0.8939 | 0.8875 | 0.3268 – 2.410 | 0.8148 |
| Sex  （Male / Female) | 1.303 | 0.6799 – 2.496 | 0.4254 | 1.469 | 0.7216 – 2.992 | 0.2888 |
| Location of metastasis  (Liver only / The others) | 1.021 | 0.4645 – 2.243 | 0.9592 | 1.035 | 0.3986 – 2.690 | 0.9430 |
| Colorectal cancer location  (Right / Left) | 0.9614 | 0.4469 – 2.068 | 0.9198 | 0.7104 | 0.2616 – 1.929 | 0.5024 |
| Treatment regimen  （SOX+C-mab / SOX+B-mab） | 1.489 | 0.7736 – 2.866 | 0.2334 | 1.622 | 0.8096 – 3.249 | 0.1725 |

HR: Hazard ratio, CI: Confidence interval
